# Economy, Movement Dynamics, and Muscle Activity of Human Walking at Different Speeds

**DOI:** 10.1038/srep43986

**Published:** 2017-03-08

**Authors:** P. C. Raffalt, M. K. Guul, A. N. Nielsen, S. Puthusserypady, T. Alkjær

**Affiliations:** 1 Department of Neuroscience and Pharmacology, University of Copenhagen, Denmark; 2 Department of Electrical Engineering, Technical University of Denmark, Denmark.

## Abstract

The complex behaviour of human walking with respect to movement variability, economy and muscle activity is speed dependent. It is well known that a U-shaped relationship between walking speed and economy exists. However, it is an open question if the movement dynamics of joint angles and centre of mass and muscle activation strategy also exhibit a U-shaped relationship with walking speed. We investigated the dynamics of joint angle trajectories and the centre of mass accelerations at five different speeds ranging from 20 to 180% of the predicted preferred speed (based on Froude speed) in twelve healthy males. The muscle activation strategy and walking economy were also assessed. The movement dynamics was investigated using a combination of the largest Lyapunov exponent and correlation dimension. We observed an intermediate stage of the movement dynamics of the knee joint angle and the anterior-posterior and mediolateral centre of mass accelerations which coincided with the most energy-efficient walking speed. Furthermore, the dynamics of the joint angle trajectories and the muscle activation strategy was closely linked to the functional role and biomechanical constraints of the joints.

The inherent intra-subject variability in human movements (i.e. cyclic movements like walking and running and repeated discrete movements like reaching or throwing) has attracted growing interest within motor control and motor learning research during the past four decades. Movement variability has been linked to sport performance level, skill acquisition, motor control related pathology and ontogenies^1^,^2^,^3^,^4^,^5^. The variability may originate from several sources such as the muscles, joints, soft tissue and central nervous system^6^,^7^. Thus, when the muscles are activated in order to produce specific movements multiple processes are initiated, which all can be potential sources to the variation in the final movement execution.

Dynamical system theory is a mathematical approach to explain the behaviour of complex systems including biological systems like the human motor control system^8^,^9^. Dynamical system theory suggests that the multiple variables within the human system (termed degrees of freedom) that are involved in the formation of movements interact through a process termed self-organization in the creation of a coordinated and stabile movement pattern (termed attractor state)^1^,^10^,^11^. An attractor can be characterized by both the spatial and temporal properties of the exhibited variability^4^,^12^. In relation to human walking, a U-shaped relationship between walking speed and the statistical persistency properties of the stride-to-stride time intervals has been observed^13^,^14^,^15^. A local minimum in the statistical persistence of time stride intervals has been shown to coincide with the self-selected preferred walking speed^16^,^17^. In line with this, it is well established that the metabolic cost of walking is minimized at the preferred walking speed and increased at speeds above and below reflecting a U-shaped relationship between walking speed and walking economy^18^,^19^.

Previous studies have separately investigated the relationship between walking speed and the rate of trajectory divergence of joint angles and centre of mass movements using the largest Lyapunov exponent^20^,^21^. England and Granata^20^ showed an increase in the rate of divergence of trajectories measured by the largest Lyapunov exponent of the ankle, knee and hip joint angles with increasing speed. While Dingwell and Marin^22^ and Kang and Dingwell^23^ showed increasing divergence of the trajectories of the centre of mass displacement with increasing walking speed Bruijn *et al*.^21^ showed that this relationship was influenced by the movement direction. Stenum *et al*.^24^ later showed that this discrepancy could be a result of methodological differences.

In agreement with the motor control theory proposed by Bernstein^25^, these previous observations indicate that the dynamics of movements are not only dependent on the task related constraints (e.g. walking speed) but also on the biomechanical constraints of the investigated structure (e.g. joints and centre of mass). However, based on the aforementioned studies it is still an open question if the relationship between the joint angle and centre of mass movement dynamics in terms of trajectory divergence and dimensionality and the walking speed resembles the observed U-shaped relationship between walking economy and speed. Furthermore, muscle activity during locomotion has been extensively investigated in order to explain the underlying motor control (e.g. reflex modulation and motor programs)^26^,^27^,^28^,^29^,^30^,^31^. However, it remains unknown if the movement dynamics and muscle activity strategy at different walking speeds exhibit the same pattern.

Several different nonlinear analyses can be used to investigate the spatiotemporal structure of human variability with each method assessing different characteristics of the time series in question^32^. The present study applies both the largest Lyapunov exponent (LLE) and correlation dimension (CoD). The LLE quantifies the exponential convergence or divergence of trajectories with time in state space and has been considered to be a measure of the local dynamic stability^4^,^33^. The CoD quantifies the fractal dimensionality of the time series and has been used to quantify how a system is organized in state space^2^,^34^. Thus, the methodological coupling of the LLE and CoD enables a detailed description of the dynamics of a system^2^,^5^. By applying this methodological approach in the present study we seek a more refined understanding of the movement dynamics of human walking at different speeds.

Thus, the purpose of the present study was to investigate the relationship between movement dynamics and walking speed. We assessed movement dynamics of the knee and ankle joint angles and the centre of mass accelerations at different walking speeds ranging across the speed considered as the most energy efficient. As mentioned previously, the U-shape relationship between walking speed and walking economy is well established in the literature. The present study measured walking economy to verify this relationship. In addition, the muscle activity pattern was assessed to evaluate how the movement dynamics is associated with the underlying motor control.

## Materials and Methods

### Participants

Twelve healthy male participants with a mean (±SD) age of 25.8 years (±5.0), height of 1.85 m (±0.07) and mass of 79.0 kg (±7.9) participated in the present study. Only male participants were recruited to reduce the variation in body height and leg length. The participants had no diagnosed lower limb injuries within the past year or neurological pathology. They were informed of the experimental conditions and gave their written consent to participate in the study. The study was approved by the local ethics committee for the Capitol Region of Denmark (in Danish: “De Videnskabsetiske Komiteer for Region Hovedstaden”), and the study was carried out in accordance with the approved guidelines.

### Protocol

The study consisted of two experimental days. On the first day, standing anthropometrics, preferred walking speed (PWS), electromyography (EMG) and joint kinematics were measured during walking at five different speeds. On the second day, oxygen uptake (VO_2_) was measured at identical walking speeds as applied on the first day. Upon arrival to the laboratory on the first day the length of both legs of each participant was measured as the distance from the trochanter major to the lateral malleolus and averaged across both legs. Nondimensionalization of walking speeds through calculation of the Froude speed (FS) for each subject was completed in order to normalize the walking speed in relation to leg length. This theoretical prediction of the preferred walking speed (TPWS) was calculated as 42% of FS (equation 1)^20^.


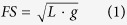


L = length leg, g = 9.82 m/s^2^.

EMG electrodes, foot switch and electro-goniometer were placed on the participants (see below) before a 5 minutes warm up walking session at a self-selected pace was completed. This was followed by a standardized procedure of isometric maximal voluntary contractions (MVC) for the knee extensor and flexor muscles and the ankle plantar and dorsiflexor muscles. During the knee extension and flexion the participants were sitting upright with 100° hip flexion and 110° knee flexion (180° = full hip/knee extension). The waist, thigh, and ankle were securely fastened to the seat. During the plantar flexion, the participants were standing upright on one leg facing a wall bar. The participants took a firmly grip of the lowest reachable bar while they remained upright and their knees fully extended which prevented heel raise and enabled an isometric contraction of the planter flexor muscles. During maximal dorsiflexion, participants were seated with their back against a wall, with 90° of knee flexion, 10° of ankle plantar flexion (0° = neutral standing position) and with the heels placed on the floor. The foot position was manually fixed by the researcher during the contraction. For all four MVCs, maximal effort was kept for three seconds under verbal encouragement. After a brief rest period, the procedure of determining the PWS was conducted following the description by Dingwell and Marin^22^. The PWS was determined by gradually decreasing and increasing the speed three times below and above the speed each participant reported as being the most comfortable. The speed display was blinded to the participants and the speed reported as being the most comfortable was noted. The average of the six noted speeds was calculated and reported as the PWS for each participant. The PWS was determined to check if this differed significantly from the TPWS.

The participants wore a pair of light weight gymnastic shoes and walked for 12 minutes on a treadmill (HS-1200 TechnoGym, Italy) at 20, 40, 100, 160 and 180% of their individual TPWS in randomized order, which provided 180 to 700 strides. Between each walking speed, the participants had between 1–4 minutes of rest to avoid fatigue.

On the second day, seven participants reported to the laboratory and completed the same warm up protocol and wore the same shoes as on the first day. The participants was fitted with the oxygen uptake face mask, connected to the oxygen uptake recording device (see below) and walked for 6 minutes at 20, 40, 100, 160 and 180% of their individual TPWS in randomized order separated by 1–4 minutes of rest.

### Measurements

Sagittal ankle and knee joint angles were measured using an electro-goniometer (Penny & Giles, M-series) placed on the lateral side of each joint. The 3D centre of mass acceleration was estimated by a tri-axial accelerometer (Marq-Medical, Farum, Denmark) placed on the sacrum fastened with sports tape. The goniometer and accelerometer were connected to a small unit containing an amplifier and A/D converter (20 × 10 × 5 mm) which was attached to the skin. The goniometer and accelerometer sensors had built-in low pass filters of 100 Hz and 290 Hz, respectively, and both sensors were an integrated part of the system used for the EMG recordings (see below).

Bipolar surface EMG electrode (2DT2 Foam Dual Pregelled Electrode, Multi Biosensors Inc., USA) were mounted on the m. soleus (SOL), m. tibialis anterior (TA), m. vastus medialis (VM), and the m. biceps femoris (BF) according to the recommendation of Perotto^35^. Micro-switches were placed under the heel and toe to record the heel strike and toe-off events of walking. Wireless EMG sensors (MQair, Marq-Medical, Farum, Denmark) were attached to the bipolar electrodes placed on SOL, TA, VM, BF muscles. Each EMG sensor consisted of an amplifier (frequency response: 10–1000 Hz) and A/D converter (16 bit). In addition, signals from the micro-switches placed under the heel and toe were also sampled by this system. Thus, the EMG, foot switch, accelerometer and goniometer signals were sampled synchronously at frequencies of 1000 Hz (EMG, foot switches) and 333 Hz (goniometer/accelerometer), respectively. The signals were wirelessly transmitted and recorded by an antenna connected to the USB port on a laptop. The signals were recorded by the software system Fireworks 1.1.0.35 (Marq-Medical, 2012, Farum, Denmark). The recordings were triggered by the foot switch placed under the heel.

The oxygen uptake was continuously measured using an Oxycon Pro breath by breath gas analysing system (Oxycon Pro, Jaeger, Wuerzburg, Germany) averaging the oxygen uptake every 5 second. Prior to the experiment, the oxygen uptake system was calibrated to the oxygen and carbon dioxide content of a known gas. The oxygen uptake data recorded during each of the 6 minutes walking trial was extracted for offline analysis.

### Data analysis

The initial 90 seconds and final 30 seconds of each joint angle signals and the 3D acceleration signals were discarded and the remaining time series were down sampled to 50 Hz. To quantify the variability of each variable the data from 150 individual strides was time normalized (0–100%). Standard deviation was calculated across all strides at each time point and averaged over the normalized stride in accordance with Dingwell and Marin^22^ as a measure of the mean variability (MeanSD).

To investigate the movement dynamics each of the down sampled time series included exactly 150 strides but with a different number of data points as done by Myers and colleagues^36^. This methodological approach maintains the same number of data points/second for all subjects and walking speeds. An alternative approach is to resample each stride to 100 data points as done by England and Granata^20^. To investigate the consequence of this methodological choice, the LLE for the knee and ankle joint was calculated using both a fixed sampling frequency and a resampling approach. Based on the results from this additional analysis presented in the Supplementary material, we concluded that the methodological choice had a limited effect on the results presented in this study. For each participant, the dynamics of the time series was investigated by calculating the LLE and CoD for the two joint angles and the three acceleration time series obtained at each speed. The LLE measures the divergence or convergence of trajectories of a time series in its state space^33^. A time series characterized by a random spatiotemporal structure within the state space with rapid diverging data points will have a relatively high LLE. The CoD is a measure of the organization of data points in state space and approximates the fractal dimension of the region in the state space occupied by the dynamical system in question^5^,^34^. A time series characterized by a high fractal dimensionality will have a relatively high CoD. In the present study, the algorithms from Wolf *et al*.^33^ and Grassberger and Procaccia^34^ were used to calculate the LLE and the CoD, respectively. The calculation procedure of these algorithms has been described in detail elsewhere^33^,^34^,^37^.

**Figure 1:**
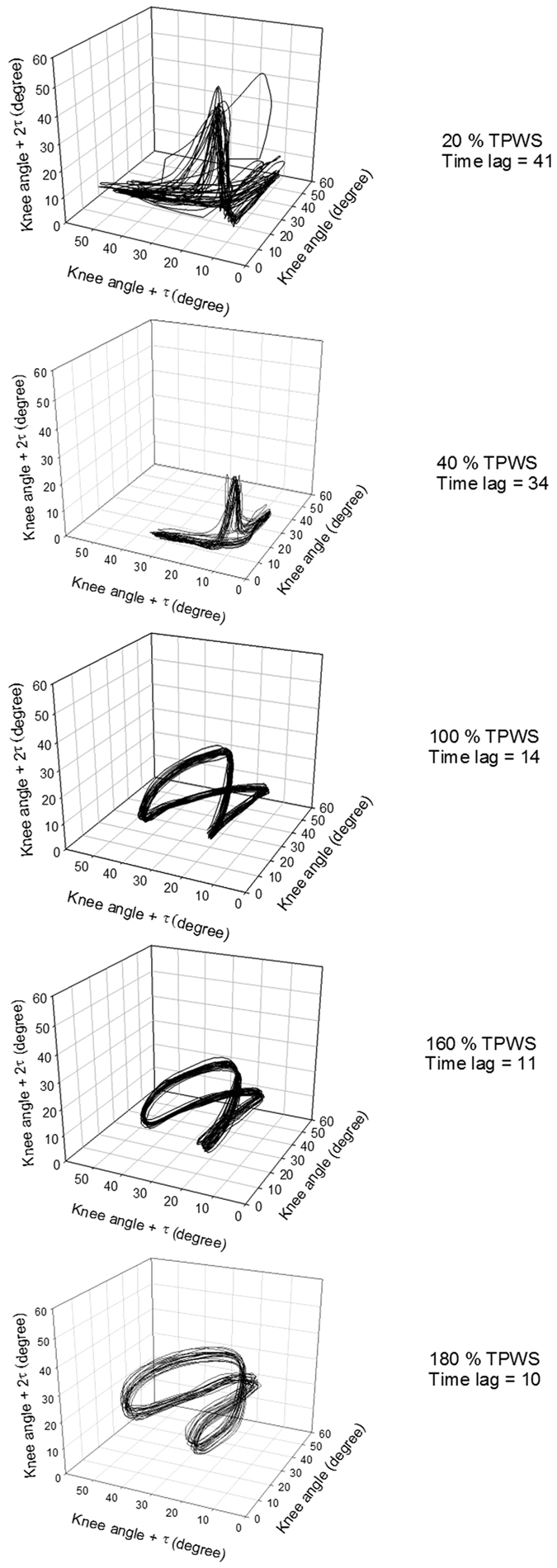
A representation of trajectories from 20 strides from one subject of the knee joint angle for each speed (top = slowest, bottom = fastest) folded in three dimensions with corresponding time lag.

In order to calculate the LLE and CoD, the time series were reconstructed in the state space using the method of delay embedding^38^,^39^,^40^. Figure 1 illustrates the organization of a time series in the state space, showing the trajectories from 20 strides of the knee joint angle at the five speeds, for one representative participant folded in three dimensions with the corresponding time lag. As previously done by Dingwell and Cusumano^4^ and Buzzi *et al*.^2^ the time delay (Tau) and embedding dimension (Dim) for delay embedding were calculated for each participant and each speed using the Average Mutual Information and False Nearest Neighbour algorithm, respectively. The group average of time delay and embedding dimension for each speed were calculated and used for the calculation of the LLE and CoD. The average, standard deviation, and range of both the time delay and embedding dimension are presented in the Supplementary material. There was a general tendency to a higher embedding dimension and time delay at speeds below 100% TPWS and constant values at 100% TPWS and above. Due to a small inter-participant variation in embedding dimension and time delay but a relatively large inter-speed variation, group averaged values were used for each speed. To support this methodological choice, LLE for the knee and ankle joint was calculated with both a group averaging of Tau and Dim and with the individual Tau and Dim. The results are presented in the Supplementary material.

Surrogate analysis is a commonly used technique applied to evaluate the nature of the fluctuation in a time series. It includes a test of the null hypothesis that fluctuations in a time series could originate from a linearly auto-correlated Gaussian process^41^. The present study applied surrogate analysis to the measured variables across the applied walking speeds^2^,^4^,^42^. For each original time series, 19 surrogate time series were constructed using the pseudo periodic method presented by Small^43^. This surrogate method was shown to be more valid with joint angle time series, which has an inherent periodic pattern compared to the Theiler’s surrogate algorithm^41^,^42^,^44^. The LLE and CoD were calculated for each of the 19 surrogate data series. The mean value of the LLE and CoD from the surrogate data was compared with that of the original time series.

The muscle activity strategy was quantified by calculating the mean muscle activity and levels of co-contraction. The recorded EMG signals were transferred to Matlab for further treatment. The signals were digitally high- and low-pass filtered (Butterworth fourth-order zero-lag digital filter, cut-off frequencies 20 Hz and 500 Hz, respectively), full-wave rectified, and low pass filtered at 15 Hz to produce linear envelopes. The linear envelopes were normalized to the maximal EMG amplitude of each muscle recorded during the MVCs.

The signal from the micro-switch on the heel was used to identify the heel strike (HS). The linear envelopes were used to calculate a co-contraction index (CCI)^45^ around the HS with a window of 100 ms before HS and 200 ms after for each of the ankle and knee joint muscle pairs (TA/SOL and VM/BF). The CCI was calculated according to the procedure described by Lewek *et al*.^46^ (equation 2):


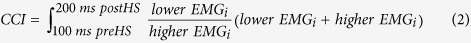


CCIs were calculated for 150 strides for each participant and a mean CCI for each of the muscle pairs was calculated and used as the input parameter for the statistical analysis.

The oxygen uptake data during the last three minutes of each walking trial was averaged and the walking economy was calculated by normalizing the oxygen uptake to the body mass and then subsequently normalizing this to the walking speed (ml O_2_/min/kg/m/min = ml/kg/m) to render the cost of movement comparable at different speeds as done by Foster and Lucia^47^.

### Statistical analysis

Paired Student’s *t*-test was used to determine if the estimated 100% TPWS was significantly different from the self-selected PWS. A one-way ANOVA for repeated measures was applied to investigate the effect of speed (independent factor) on the dependent variables of MeanSD, LLE and CoD for knee and ankle angles and centre of mass accelerations, EMG, co-contraction index and walking economy. In case of a significant effect of speed, a quadratic regression analysis was performed to determine the nature of the relationship between the walking speed and dependent measure. P-values and the overall percentage of variance accounted for by the regression (r^2^) were determined for each dependent variable. Furthermore, a Holm-Sidak post hoc test was applied to evaluate the between-speed differences. The level of significance was set to 5%. In total, 1140 surrogate time series for each variable (5 speeds × 12 participants × 19 surrogate time series) were created. For each participant, all variables at each speed were compared to the 95% confidence interval of the corresponding surrogate variable. All statistical analyses were performed using the Sigmaplot (Systat Software, Inc. 2014) and Matlab (MathWorks R2011b).

## Results

### Theoretical preferred walking speed (TPWS) and preferred walking speed (PWS)

An average PWS of 4.69 km/h (SD = 0.47 km/h) was observed and this was not significantly different from the 100% TPWS of 4.43 km/h (SD = 0.09 km/h) (p = 0.108).

### MeanSD of joint angles and body mass acceleration

**Figure 2:**
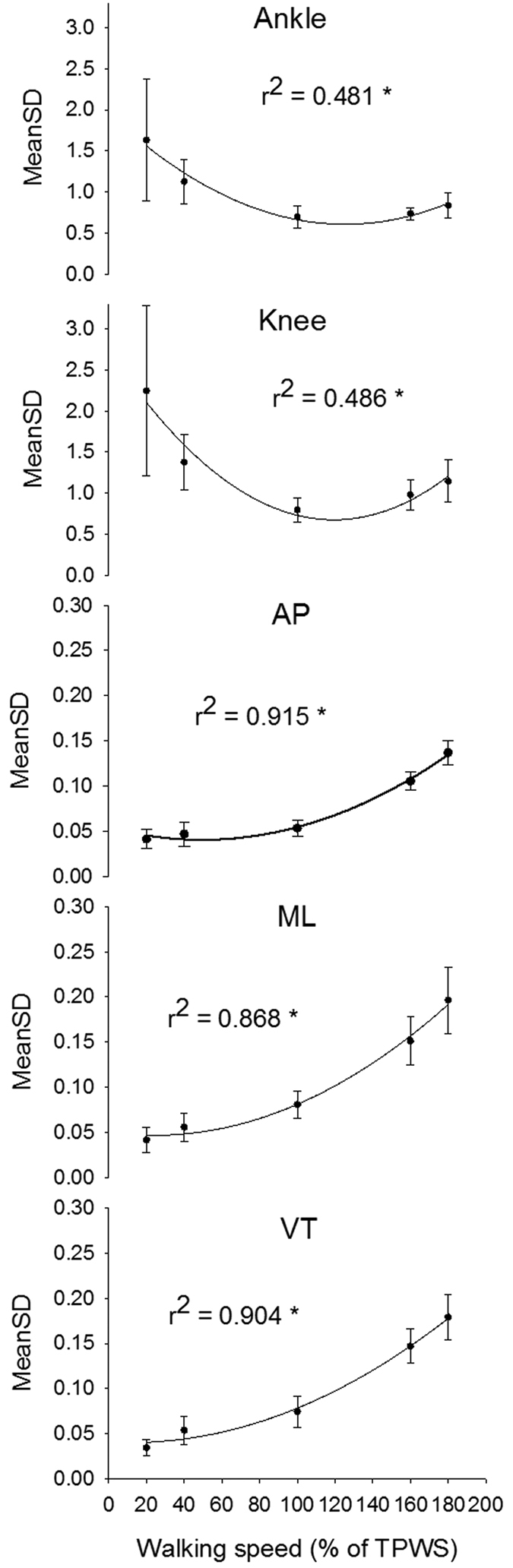
MeanSD for the ankle and knee joint angles and the AP, ML and VT centre of mass accelereation. Error bars indicate between-subject standard deviation. Curve fit lines represent quadratic polynomial regression curves with r^2^ values indicate the percent of variance accounted for by each regression model. *Indicates significant quadratic relationship between walking speed and variable.

There was a significant effect of speed on the MeanSD for both joint angles (p < 0.001 in both cases). The quadratic curvilinear fit was also statistically significant (p < 0.001 in both cases) and walking speed could explain 48% of the variance (Fig. 2). The MeanSD of both joint angles were significantly higher at 20% TPWS compared to higher walking speeds (p < 0.001 for all comparisons). The ankle MeanSD at 40% TPWS was significantly higher compared to 100% and 160% TPWS (p = 0.01 and p = 0.019, respectively) but did not differ from 180% TPWS. The knee MeanSD at 40% TPWS was significantly higher compared to 100% TPWS (p = 0.022) but did not differ from higher walking speeds. No other between-speed differences were observed for the ankle and knee MeanSD.

There was a significant effect of speed on the MeanSD for the centre of mass acceleration in all directions (p < 0.001 in all cases). The quadratic curvilinear fit was significant (p < 0.001 for all three fits) and showed an increasing pattern in MeanSD with increase in walking speed. Walking speed could explain 86% to 91% of the variance (Fig. 2). The post hoc test showed no significant difference in MeanSD for the anterior-posterior and mediolateral direction between 20% and 40% TPWS and between 40% and 100% TPWS for the anterior-posterior direction. All other between-speed comparisons showed a significant difference (p ≤ 0.014 for all comparisons).

### Nonlinear analysis of joint angles

**Figure 3:**
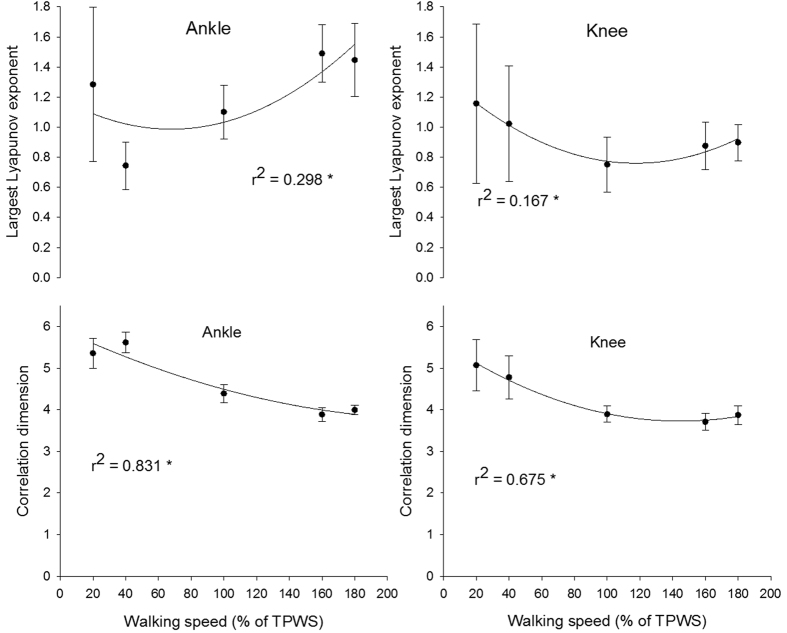
Largest Lyapunov exponent and correlation dimension of the ankle and knee joint angle. Error bars indicate between-subject standard deviation. Curve fit lines represent quadratic polynomial regression curves with r^2^ values indicate the percent of variance accounted for by each regression model. *Indicates significant quadratic relationship between walking speed and variable.

There was a significant effect of walking speed on the LLE and CoD of the ankle joint angle (p < 0.001 for both angles). The quadratic curvilinear fit was significant for both the LLE and CoD (p < 0.001) and the walking speed explained 29% and 83% of the variance, respectively (Fig. 3). The LLE at 40% TPWS was significantly lower compared to the higher walking speeds (p ≤ 0.018 for all comparisons). At 100% TPWS the LLE was significantly lower than at 160% and 180% TPWS (p = 0.009 and p = 0.02, respectively). No other between-speed differences were observed for the LLE of the ankle joint angle. There were significant differences for all between-speed comparisons for the CoD of the ankle joint angles (p ≤ 0.003 for all comparisons) except between 160% and 180% TPWS.

There was a significant effect of walking speed on the LLE and CoD of the knee joint angle (p = 0.009 for LLE and p < 0.001 for CoD). The quadratic curvilinear fit was significant (p = 0.005 for LLE and p < 0.001 for CoD) and walking speed explained 16% and 67% of the variance in the LLE and CoD, respectively (Fig. 3). The LLE of the knee joint angle was significantly lower at 100% TPWS compared to 20% TPWS (p = 0.007). No other between-speed differences were observed. The CoD at 20% and 40% TPWS was significantly higher compared to the higher walking speeds (p < 0.001 for all comparisons). No other statistically significant differences were observed.

### Nonlinear analysis of centre of mass acceleration

**Figure 4:**
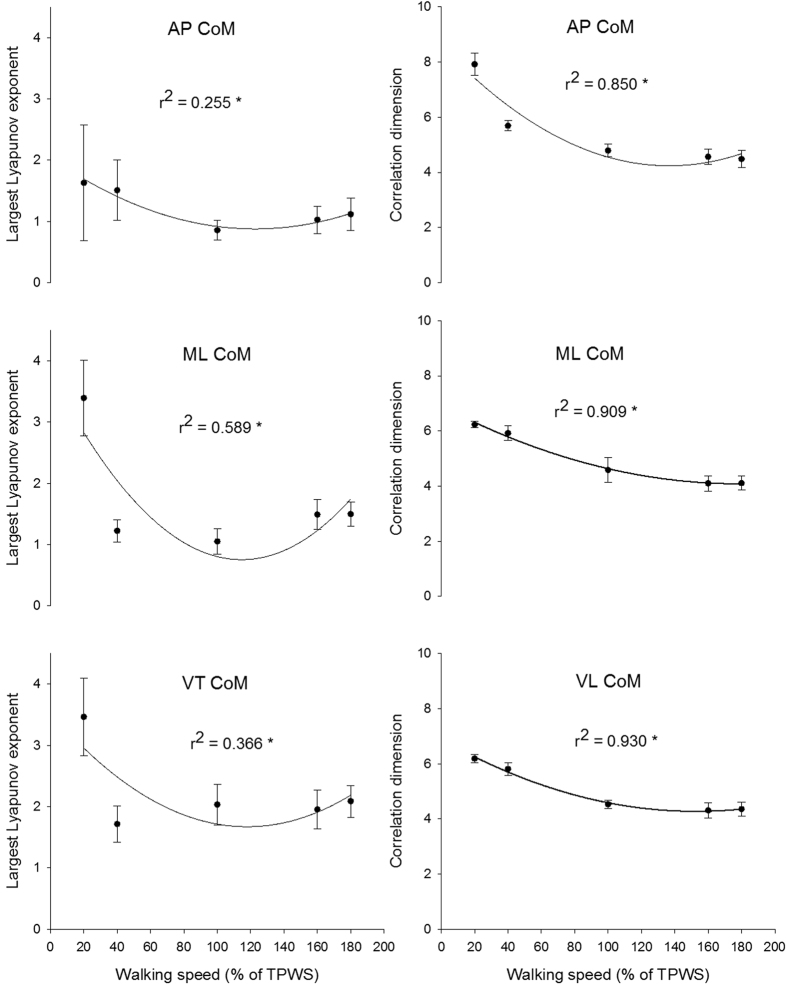
Largest Lyapunov exponent and correlation dimension for anterior-posterior, medio-lateral and vertical direction of centre of mass accerelation. Error bars indicate between-subject standard deviation. Curve fit lines represent quadratic polynomial regression curves with r^2^ values indicate the percent of variance accounted for by each regression model. *Indicates significant quadratic relationship between walking speed and variable.

There was a significant speed effect for both the LLE and CoD of the centre of mass accelerations in all three directions (p < 0.001). The quadratic curvilinear fits were also significant (p < 0.001 in all cases) and walking speed explained between 25% and 58% of the variance for the LLE and between 85% and 93% of the variance for the CoD (Fig. 4). The LLE of the anterior-posterior centre of mass acceleration was significantly higher at 20% TPWS compared to 100 and 160% TPWS (p = 0.005, 0.042, respectively) and higher at 40% TPWS compared to 100% TPWS (p = 0.025). The CoD of the anterior-posterior centre of mass acceleration was significantly higher at the two lowest walking speeds compared to the faster walking speeds (p < 0.001 in all cases). In addition, the CoD at 100% TPWS was significantly higher compared to 180% TPWS (p = 0.0016). No other statistically significant between-speed differences were observed.

The LLE of the mediolateral centre of mass acceleration was significantly higher at the lowest walking speed compared to the higher walking speeds (Fig. 4, p < 0.001 in all cases). Furthermore, the LLE was significantly lower at 100% TPWS compared to the two higher walking speeds (p = 0.007 in both cases). The CoD of the mediolateral centre of mass acceleration decreased significantly with each increment in walking speed (p ≤ 0.013 in all cases) except from 160% to 180% TPWS (Fig. 4).

The LLE of the vertical centre of mass acceleration was significantly higher at the lowest walking speed compared to the higher walking speeds (p < 0.001 in all cases). Furthermore, the LLE at 40% TPWS was significantly lower than at 180% TPWS (p = 0.003). The CoD of the vertical centre of mass acceleration decreased significantly with each increment in walking speed (p ≤ 0.013 in all cases) except from 160% to 180% TPWS.

### Surrogate analysis

The surrogate analysis of the joint angles showed that 100% of the LLE calculated on the original ankle joint angle time series were lower than the 95% confidence interval of the LLE calculated on the corresponding surrogates. For the knee joint angle, 91.7% of the LLE calculated on the original knee joint angle time series were lower than the 95% confidence interval of the LLE calculated on the corresponding surrogates. When comparing the CoD calculated on the original and corresponding surrogates, the CoD from 43.3% of the ankle joint angle time series and from 46.7% of the knee joint angle time series were lower than the 95% confidence interval of the CoD from the surrogates.

The surrogate analysis of the centre of mass accelerations signals showed that between 98.3% and 100% of the LLE calculated on the original time series were lower than the 95% confidence interval the LLE from the corresponding surrogates. Equally, between 95% and 100% of the CoD calculated on the original centre of mass acceleration time series were lower than the 95% confidence interval of the CoD calculated on the corresponding surrogates.

### Muscle activity

**Figure 5:**
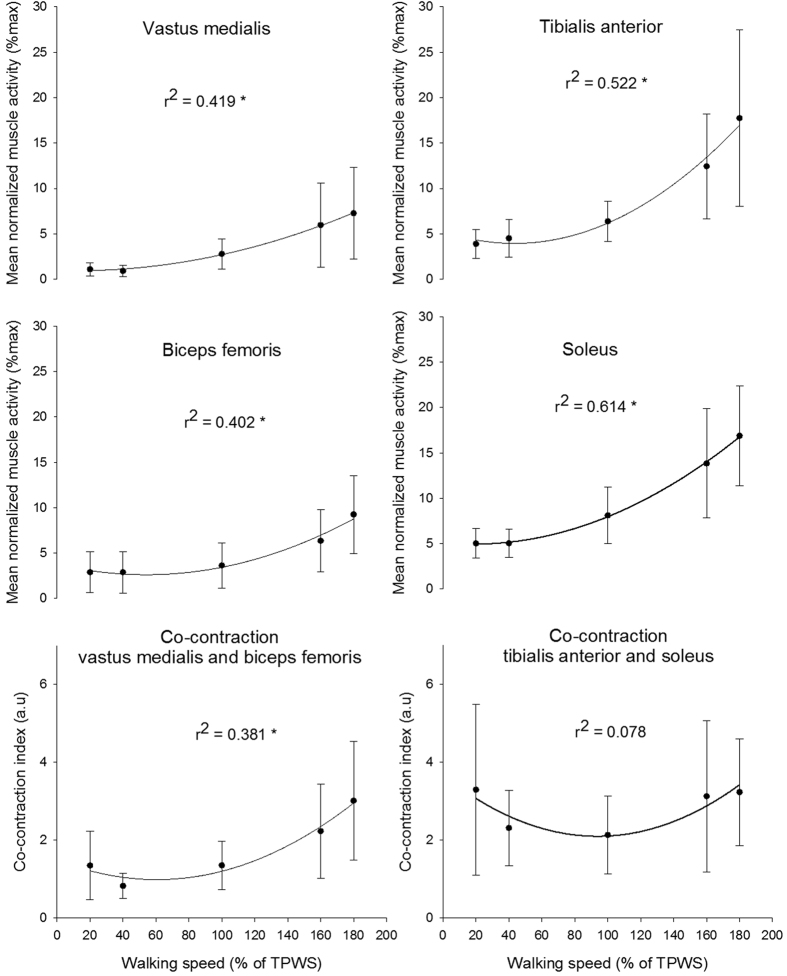
Muscle activity and co-contraction index for each muscles and muscle pairs. Error bars indicate between-subject standard deviation. Curve fit lines represent quadratic polynomial regression curves with r^2^ values indicate the percent of variance accounted for by each regression model. *Indicates significant quadratic relationship between walking speed and variable.

There was a significant effect of speed on the muscle activity level of all four muscles (p < 0.001). The quadratic fit was also significant for all four muscles (p < 0.001) and walking speed explained between 40% and 61% of the variance (Fig. 5). The VM activity was significantly higher at 160 and 180% TPWS compared to the three lower walking speeds (p ≤ 0.005 for all comparisons) but did not differ significantly between the three lowest or between the two highest walking speeds. The muscle activity of both BF and TA were significantly higher at the two highest walking speeds compared to the three lower walking speeds (p < 0.001 for all comparisons). In addition the activity was significantly higher at 180% TPWS compared to 160% TPWS. The muscle activity of the SOL increased significantly from 40% to 100% TPWS and from 100% to 160% TPWS (p ≤ 0.012 in both cases). No significant differences were observed between 20% and 40% TPWS and between 160% and 180% TPWS.

There was a significant effect of speed on the co-contraction index of the VM and BF (p < 0.001) with a significant quadratic fit (p < 0.001) and 38% of the variance was explained by the walking speed (Fig. 5). The co-contraction index was significantly higher at the two highest walking speeds compared to the three lowest walking speeds (p ≤ 0.011). Furthermore, the co-contraction index was significantly higher at 180% TPWS compared to 160% TPWS (p = 0.02). There was an overall effect of walking speed on the co-contraction index of the TA and SOL (p = 0.024). However, the quadratic fit was not statistically significant and only 7% of the variance was explained by the walking speed (Fig. 5). The post hoc test revealed no statistically significant between-speed differences.

### Walking economy

**Figure 6:**
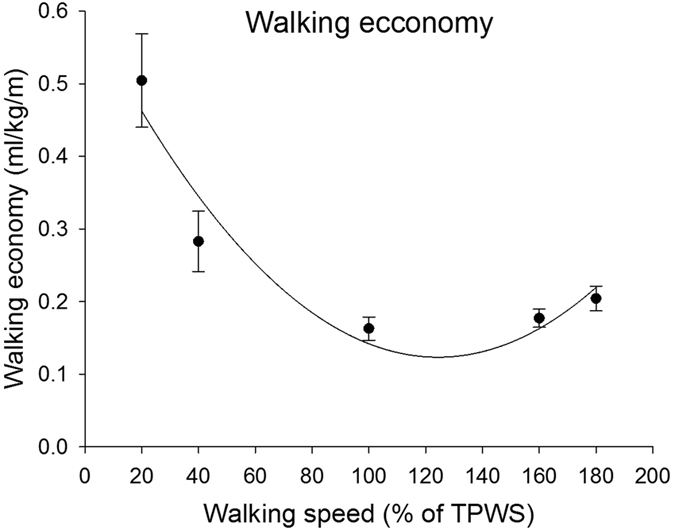
Walking economy for each walking speed. Error bars indicate between-subject standard deviation. Curve fit lines represent quadratic polynomial regression curves with r^2^ values indicate the percent of variance accounted for by each regression model. *Indicates significant quadratic relationship between walking speed and walking economy.

There was an overall effect of walking speed on walking economy (p < 0.001). The quadratic fit was significant (p < 0.001) and 85% of the variance was explained by the walking speed (Fig. 6). The walking economy was significantly higher at the two lowest walking speeds compared to the three higher speeds (p < 0.001 for all comparisons). Furthermore, the walking economy at 100% TPWS was significantly lower compared to 180% TPWS. No other between-speed differences were observed.

## Discussion

The present study aimed to investigate the relationship between the movement dynamics (assessed for the knee and ankle joint angles and the centre of mass accelerations) and walking speed. We observed an intermediate state in the dynamics of the anterior-posterior and medio-lateral centre of mass acceleration and the knee joint angle at 100% TPWS corresponding to speed where there was a minimum in walking economy. At this walking speed the LLE was relative low compared to walking speeds above and below and the CoD was in-between the values observed at speeds above and below. Regarding the ankle joint movement dynamics and muscle activation strategy, the results were more differential which may be due to differences in the anatomy and functional demands of the knee and ankle joints.

In the present study, the dynamics of knee and ankle joint angles and centre of mass accelerations was quantified by the LLE and CoD. These two measures quantify different characteristics of the time series. CoD quantifies the fractal dimensionality of the time series unfolded in the state space and provides a measure of the spatial organization based on the temporal evolution of the time series. A low value could reflect that the time series is organized in a tighter fashion compared to a high value. The LLE on the other hand quantifies the rate at which the trajectories diverge or converge in state space. Thus, it is a measure of changes in the time series when unfolded in the state space. A low value means small changes over time, and a high value means larger changes over time. The combination of CoD and the LLE provides valuable information of the structure of the time series. High CoD and LLE indicate a less organized structure with rapid changes. In contrast, low values in both parameters indicate a more organized structure with less rapid changes. These two extremes could characterize a system as either randomly or periodically structured. An intermediate (i.e. chaotic) structure has been suggested to be linked with movement attractors observed in young and healthy humans in contrast to the elderly and/or diseased^12^,^36^.

Several studies have investigated different biomechanical parameters during walking at different speeds and different methods have been used to determine the investigated speeds. Bruijn *et al*.^21^ used five absolute speeds ranging from 0.62 m/s to 1.72 m/s with an inter-speed interval of 0.22 m/s. Jordan *et al*.^14^ used five percentages of PWS ranging from 80% to 120%. In the present study, the investigated speed was determined as 42% of the FS as done by England and Granata^20^. This method provided a feasible and valid estimate of the theoretical predicted preferred walking speed which also appeared to be comparable to the self-perceived PWS reported by the participants.

The variability in joint angles and centre of mass acceleration was measured by MeanSD across all 150 strides. Thus, MeanSD quantifies the average amount of variation in joint movements and centre of mass acceleration within each stride but does not take into account the order of strides. MeanSD can be considered to be a measure of the consistency where low values indicate high consistency and high values indicate low consistency. The joint angles and centre of mass accelerations showed opposite patterns. The consistency of the joint angles was low at the lowest speeds and high at the highest velocities which confirms previous observations^48^. In contrast, the consistency of the centre of mass accelerations in all directions decreased with walking speed. The walking speed dependency of the joint angle variability observed in the present study resembles that observed by Jordan and colleagues on the normalized variability (measured as coefficient of variance) in stride length and step length^14^. Based on this, it could be speculated that the consistency in lower limb joint movements in the sagittal plane is closely related to the consistency of step and stride length. In the same study, the authors observed an increase in ground reaction force consistency with walking speed which is well in line with the increasing centre of mass acceleration variability across speeds observed in the present study^14^. With respect to the movement of the centre of mass, the results of the present study are in contrast to other observations^49^. Dingwell and Marin reported a significant quadratic curvilinear fit between walking speed and the MeanSD of the velocity of the upper body (the first thoracic vertebrae) where between 11% and 28% of variance could be explained by the regression model and with a local minimum in MeanSD at PWS^49^. The present study could not confirm the U-shaped relationship suggested by the authors of the aforementioned study. This discrepancy could be explained by methodological differences.

The dynamics of the joint angles was strongly dependent on both the joint in question and the changes in speed. The CoD of both joints decreased with increase in walking speed, indicating a more tightly organized structure at higher speeds. The LLE of the knee joint angle showed tendency to a U-shape relationship indicating rapid changes in the trajectories at both lower and higher speeds. This is visualized in Fig. 1, where the knee joint angle at the slowest speeds reflected a relatively random pattern with limited structure. At 100% TPWS the knee joint angle trajectory exhibited a more distinct pattern with a tight organization. This pattern became less smooth and with more rapid changes with increasing speed.

A different pattern was observed for the ankle. The LLE at 20% TPWS was significantly higher when compared to 40% TPWS but the CoD at these speeds did not differ. This indicates that both walking conditions had relatively unorganized structures in the state space but the slowest speed induced much more rapid changes in the trajectories. This is potentially due to the functional characteristics of the ankle joint, and the differences in the movement task solved by the joint. During one-legged balance, a large portion of movement control of the centre of mass is located at the ankle joint^50^,^51^ and the subtalar joint is partly responsible for the medio-lateral movements. It is likely that during the slowest speed, the task of maintaining medio-lateral balance in the subtalar joint interferes with the dorsi/plantar-flexion of the talocrural joint, resulting in rapid changes in the trajectories quantified by the relatively high LLE value. As speed increases, the challenge to maintain medio-lateral balance decreases, which may explain the reduced LLE in the sagittal plane. As the speed increases above 100% TPWS, the impact increases, and due to its role as the first major shock absorber in the leg; rapid changes will be induced to the ankle joint angle trajectories. Our joint angle results are partly in agreement with the results presented by England and Granata^20^. Within the comparable speeds used by both England and Granata^20^ and the present study, the LLE for the ankle joint angle showed the same tendency to an increase with increasing speed. However, the increase in LLE for the knee joint angle observed by England and Granata^20^ could not be confirmed by the present study. This discrepancy could possibly be explained by differences in the applied embedding dimensions, the algorithm to estimate the LLE, number of strides and the normalization technique^24^.

When comparing the dynamics of the centre of mass accelerations, the LLE for the anterior-posterior direction best resembled the U-shaped curve observed in walking economy. When combined with decay in the CoD, the anterior-posterior acceleration at 100% TPWS was characterized by a relative tight organization with slower changes. At a lower speed, the acceleration seemed to become less tightly organized and with more rapid changes and at a higher speed, the rate of divergence increased again. The medio-lateral and vertical accelerations of the centre of mass showed a slightly different relationship with speed. Although the pattern with decreasing CoD and thereby a more tightly organized structure was seen in all three directions, the LLE however, revealed differences. At the slowest speed, both the medio-lateral and vertical acceleration showed very rapid changes in the trajectories. It is possible that this is due to the difficulties of walking at a very slow speed, which in our case almost resembled a one-legged, postural balance task. This task induced many small and rapid adjustments of the position of the centre of mass not directly related to moving the centre of mass forward. At higher speeds the medio-lateral acceleration was also characterized by more rapid changes. Thus, it seemed that slow walking was linked to a decreased adaptability through a less organized structure with rapid changes while faster walking was linked to a tighter organization with rapid changes. Together, these observations indicated that the local minimum in walking economy coincided with a structure of the centre of mass anterior-posterior and medio-lateral acceleration that could be characterized as an intermediate stage somewhere between a loosely organized system with rapid changes and a very tightly organized system with rapid changes. In line with a previously presented model of human movement variability by Stergiou and colleagues, we suggest that this intermediate stage reflects an optimal condition, where the dynamics of the system provides a high adaptability and robustness towards external perturbations^12^,^36^. It should be mentioned that the dynamics of the knee joint angle had a similar intermediate stage at 100% TPWS, which could indicate that the movement of that joint angle also possess a high adaptability at that walking speed.

Bruijn *et al*.^21^ calculated the LLE on the displacement trajectories of the centre of mass during walking in a range of speeds. Three different patterns were observed for the three directions of the centre of mass. The LLE of the displacement trajectories in the anterior-posterior direction decreased with increasing speed. A tendency to an inverted U-relationship was observed in the medio-lateral direction and an increase was observed for the vertical direction with increasing speed. Additionally, Dingwell and Marin^22^ and Kang and Dingwell^23^ showed increasing divergence of the trajectories of the centre of mass displacement with increasing walking speed. We were not able to reproduce the findings reported by Bruijn *et al*.^21^ Dingwell and Marin^22^ and Kang and Dingwell^23^. This discrepancy could likely be due to methodological difference (e.g. choice of LLE algorithm and stride normalization procedure)^24^.

The muscle activity reflects the functional role of each muscle. All four muscles showed the same increasing pattern with increasing speed. However, TA and SOL increased more rapidly. With increasing speed, the need for increased propulsion is accounted for by increased activity in the SOL^52^. This may also raise the need for both rapid toe lift during the swing phase and the control of plantar flexion at heel strike, all done by TA^53^. The BF and VM were hardly active at or below 100% TPWS while the activities of these muscles were increased at higher speeds suggesting an increased need for shock absorption. This is also reflected in the co-contraction index which followed the same pattern. The co-contraction index was calculated for the period just around heel strike, where the impact of the ground contact is absorbed. For the distal joint the muscle pair furthermore needs to prepare for the balance task by increasing the joint stability^54^. Thus, we suggest that the co-contraction index reflects two tasks maintained by the ankle joint around heel strike: shock absorption and balance control at low speeds and mainly shock absorption at higher speeds. At higher speeds the proximal muscle pairs contributed substantially to the shock absorption^55^.

The present study measured oxygen uptake in order to verify that the U-shaped relationship between walking speed and walking economy previously established in young healthy individuals also existed in the participants included in the present study. This relationship could be confirmed.

Before calculating the LLE and CoD the time series are reconstructed in state space using the embedding dimension and time lag. In the present study, a group averaged embedding dimension and time lag for each speed were used as previously done^4^,^20^,^21^,^56^. An alternative approach is to use the individual determined embedding dimension and time lag for each time series. In the present study, this approach would resolve in (12 participants × 5 speeds × 5 variables) 300 unique state spaces compared to the (1 × group × 5 speeds × 5 variables) 25 state spaces used. Van Schooten and colleagues^57^ investigated the test-retest reliability of calculation of LLE from the centre of mass acceleration during walking using both the individual and group averaging approach when calculating embedding dimension and time lag for state space reconstruction. Based on intra-class correlations and smallest detectable differences the authors concluded that the group averaging approach was the most appropriate^57^. Based on the results presented in the Supplemental material where no significant differences in the LLE results were observed between the two methods, we concluded that the applied method was valid.

The LLE has been widely used to quantify the dynamics of walking in a number of studies^2^,^4^,^49^,^56^,^58^,^59^ with two dominating algorithms: the Wolf algorithm^33^ and the Rosenstein algorithm^60^. The Wolf algorithm has been proven to be more sensitive to detect age-related and joint-related differences during walking compared to the Rosenstein algorithm^37^. Based on the differences between the two algorithms described in detail by Cignetti *et al*.^37^ and Bruijn *et al*.^61^ and the discussion of the sensitivity of the algorithm in Cignetti *et al*.^37^, Bruijn *et al*.^61^ and Cignetti *et al*.^62^, we decided to use the Wolf algorithm.

Since the LLE has been shown to be sensitive to the number of strides investigated^63^, we decided to adjust the time series to contain an equal number of 150 strides for all participants and speeds. To the best of our knowledge no consensus exists regarding the normalization process of the time series used for the LLE. To avoid differences in the number of data points used in the calculation, studies have normalized time series either to the same total number of points^20^,^37^,^64^ or normalized each stride to a given number of points^20^,^49^. However, this procedure rescales the time series and could potentially hide the time dependent behaviour of the system. The aim of the present study was to investigate the effect of different walking speeds on the movement dynamics. One major change in the movement solution is a decreasing stride time with increasing speed. Thus, the number of time points available for the system to adjust the behaviour is greatly changed with increasing speed. Interpolating the time series and adding or removing data points will potentially hide behavioural characteristics of the trajectory in state space. Thus, we chose not to rescale the time series^36^,^56^,^65^. The analysis presented in the supplemental material did reveal a significant difference in the LLE of the knee but not of the ankle joint angle when comparing the results based on the applied method (fixed sampling frequency with different number of data points versus an alternative method – using normalized time series with an equal number of data points). However, the overall pattern of the LLE across the different speeds was qualitatively similar for the two methods. While this supports the chosen methodology in the present study, it should be noted that the Supplementary material did not include the centre of mass acceleration signals or the correlation dimension analyses. Thus, a potential effect of the normalization technique on these results cannot be excluded.

In the present study, walking economy was measured from a subset of the subjects and on a different day than the kinematics and muscle activity. Even though this could be considered a limitation of the present study, the presented results confirmed what has been previously reported in the literature^18^,^19^. In addition, day-to-day variations in walking economy have been shown to be modest^66^. Thus, we consider these factors to have a limited influence on the results.

In conclusion, we found that at 100% TPWS the walking economy was minimized and the dynamics of the anterior-posterior, medio-lateral centre of mass acceleration and the knee joint angle exhibited an intermediate stage. We interpret this as a stage of optimal variability of the human system meaning that it has a high degree of adaptability. This stage also appears to be the most energy-efficient. Furthermore, the dynamics of the joint angle trajectories and the muscle activation strategy were closely linked to the functional role and biomechanical constraints of the joints.

## Additional Information

**How to cite this article**: Raffalt, P. C. *et al*. Economy, Movement Dynamics, and Muscle Activity of Human Walking at Different Speeds. *Sci. Rep.* **7**, 43986; doi: 10.1038/srep43986 (2017).

**Publisher's note:** Springer Nature remains neutral with regard to jurisdictional claims in published maps and institutional affiliations.

## Supplementary Material

Supplementary Information
