# Supplementary material for: Economy, Movement Dynamics, and Muscle Activity of Human Walking at Different Speeds
Source: Sci Rep. 2017 Mar 8;7:43986. doi: 10.1038/srep43986 (PMC5341064; doi:10.1038/srep43986)
Supplement: Supplementary Information [file srep43986-s1.pdf]

SUPPLEMENTARY MATERIAL

Title:

Economy, Movement Dynamics, and Muscle Activity of Human Walking at Different Speeds

Authors:

Raffalt, PC.[1], Guul, MK.[1], Nielsen, AN.[1], Puthusserypady, S.[2], Alkjær, T.[1]

Affiliations:

[1] Department of Neuroscience and Pharmacology, University of Copenhagen

[2] Department of Electrical Engineering, Technical University of Denmark

Corresponding author:

Peter C. Raffalt

Department of Neuroscience and Pharmacology

University of Copenhagen

Email: raffalt@sund.ku.dk

Mobil phone: +45 30 56 49 66

Supplementary – Part 1

This supplementary material had two purposes:

1) To investigate the effect of the use of group averaging time delays (Tau) and embedding dimension (Dim) on calculation of the largest Lyapunov exponent (LLE).
2) To investigate the effect of the non-normalized time series on the calculation of the largest Lyapunov exponent (LLE).

The present study used a group averaging approach where Tau and Dim were averaged across all subjects for each of the five velocities. The average, standard deviation and range of Tau and Dim within the group are presented in table 1 in the manuscript. The LLE of the ankle and knee joint angels and center of mass accelerations was calculated using these average values. An alternative approach is to use the individual Tau and Dim for each subject and speed for the LLE calculation. The study by Van Schooten et al. 2013 supports the group averaging approach but this is to the best of our knowledge the only study directly addressing this methodological issue. To evaluate the difference in the LLE results based on these two methods (group averaging vs. individual Tau and Dim) LLE was calculated on the ankle and knee joint angle time series for all subjects at all walking speeds with both the group averaging approach and the individual Tau and Dim approach. The results are presented in figure S1.

The present study use an equal number of 150 strides but an unequal number of data points for each time series sampled due to a fixed sampling frequency. An alternative approach is to resample each stride to an equal number of data points in order to create time series of equal length for all subjects at all conditions. To compare these two methodological approaches (fixed sampling frequency vs. resampled) each method was applied to the ankle and knee joint angle creating two sets of time series. The LLE was then calculated of each time series (using the group averaging Tau and Dim). The results are presented in figure S2.

A two way ANOVA for repeated measures (speed x method) was applied to test the effect of choice of method (group averaging vs. individual Tau and Dim and fixed sampling frequency vs. resampled, respectively) on the calculated LLE. The level of significance was 5 % and Holm-Sidak post hoc test was applied in case of significant overall effects.

There was no effect of the choice of method when comparing the group averaging and individual Tau and Dim approach on either the ankle or knee joint angles. Based on a qualitative evaluation of the LLE from each subject in figure S1, it appears that the ankle LLE display a more distinct pattern across all subjects when calculated using the group averaging approach compared to the individual approach. This tendency is not equally clear for the knee LLE.

There was significant effect of choice of method when comparing the fixed sampling frequency and resampled method for the knee joint angle ($p=0.035$) but not for the ankle joint angle. The post hoc test revealed significant differences between the two methods at all walking speeds. At the two lowest walking speeds, the LLE calculated with the resampled method was significantly higher (20% TPWS: $p<0.001$, 40% TPWS: $p=0.015$) compared to the LLE calculated with the fixed frequency. At the three higher walking speeds, the LLE calculated with the resampled method was significantly lower compared to the LLE calculated with the fixed frequency (100% TPWS: $p=0.002$, 160% TPWS: $p<0.001$, 180% TPWS: $p=0.008$).

Based on these results, it can be concluded that using the group averaging approach compared to the individual approach for choosing Tau and Dim does not significantly affect the overall results of the calculated LLE. In contrast, the method choice regarding fixed sampling frequency or resampling influences the results of the knee joint angle LLE. While the fixed sampling frequency method used in the main study resulted in only one significant between-speed difference (between 20% TPWS and 100% TPWS), using a resampling method would presumably have resulted in an additional between-speed difference between 40% and 100% TPWS (see figure S2-F).

In conclusion, the data presented in this supplementary material validates the methodological choices made in the present study with respect to choice of Tau and Dim. However, the applied fixed frequency method diminishes the speed related changes in LLE at lower walking speeds. Accordingly, comparisons of LLE calculated with different time series normalization methods should be made with cautions. It is beyond the scope of the supplementary material to statistically favor either one of the normalization methods.

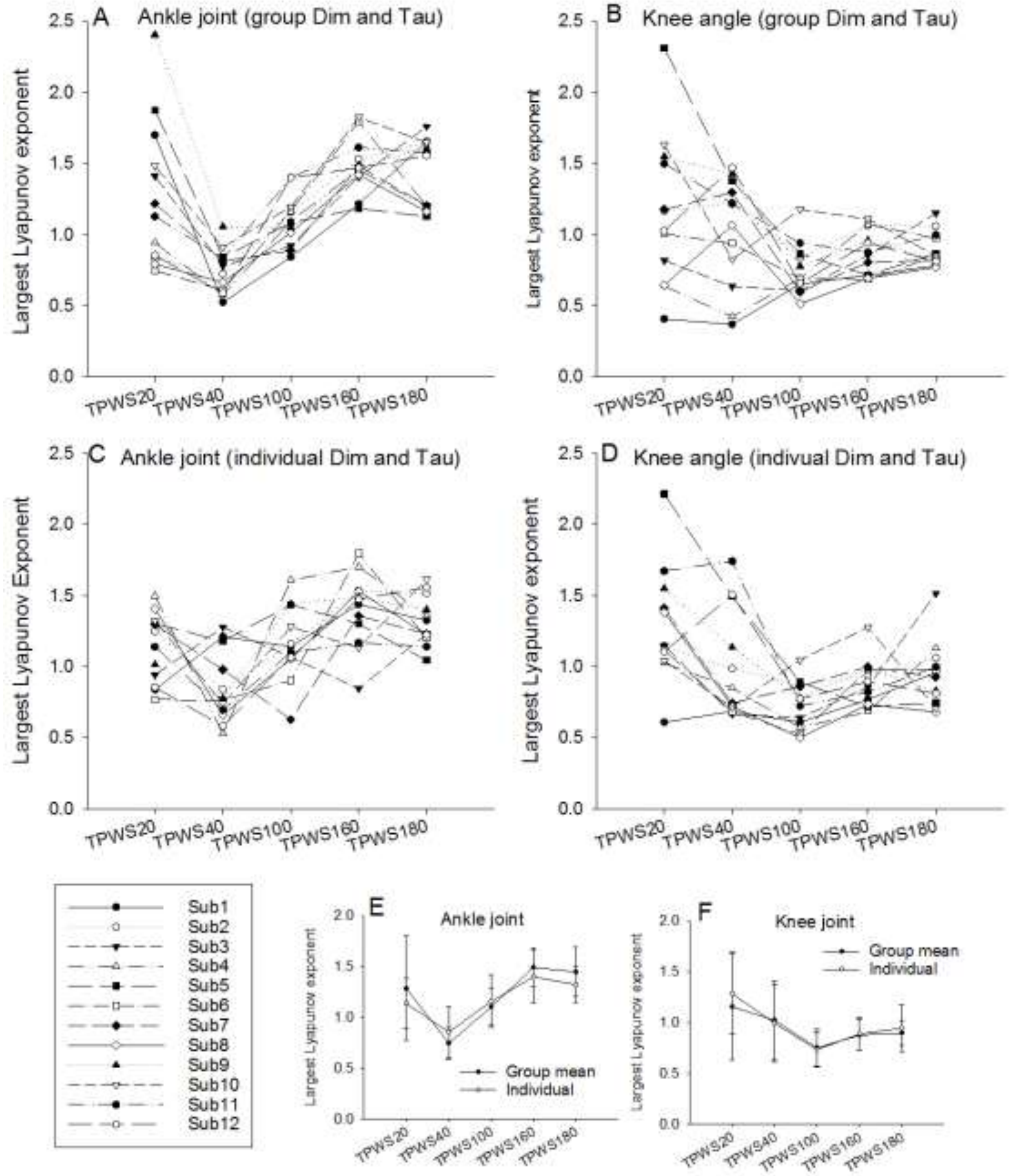

Figure S1: A) LLE for the ankle joint angles for all subjects with the group averaging approach, B) LLE for the knee joint angles for all subjects (sub1 to sub12) with the group averaging approach, C) LLE for the ankle joint angles for all subjects with the individual approach, D) LLE for the knee joint angles for all subjects with the individual approach, E) Group averaging of LLE for the ankle joint angles with the group averaging and individual approach, F) Group averaging of LLE for the knee joint angles with the group averaging and individual approach.

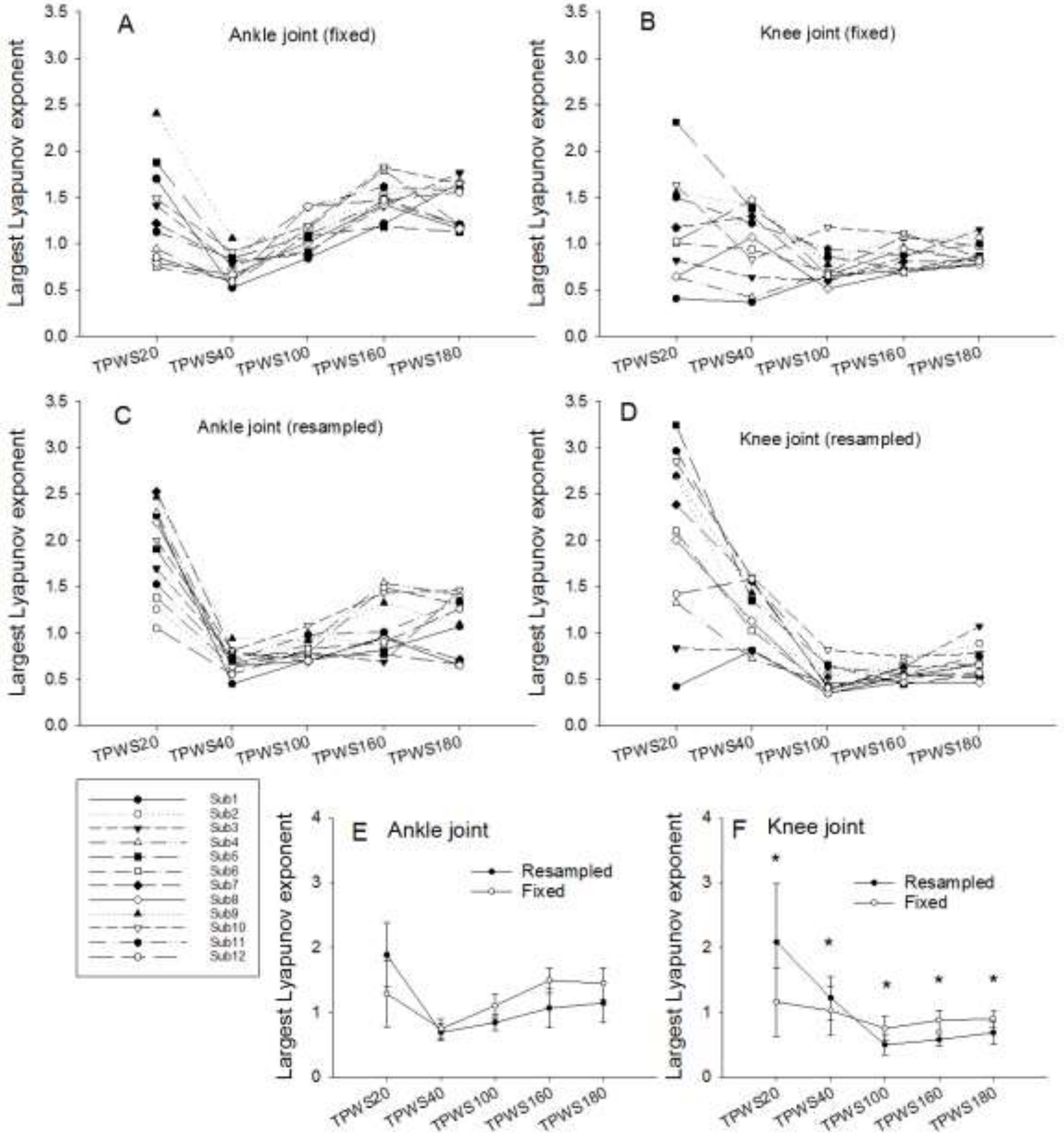

Figure S2: A) LLE for the ankle joint angles for all subjects with the fixed sampling frequency method, B) LLE for the knee joint angles for all subjects (sub1 to sub12) with the fixed sampling frequency method, C) LLE for the ankle joint angles for all subjects with the resampled method, D) LLE for the knee joint angles for all subjects with the resampled method, E) Group averaging of LLE for the ankle joint angles with the fixed sampling frequency and resampled method, F) Group averaging of LLE for the knee joint angles with the fixed sampling frequency and resampled method. * indicates significant difference between methods ($p<0.05$).

Supplementary – Part 2

Table S1

Mean, standard deviation (SD) and range of time lag (Tau) and embedding dimension (Dim) for the ankle and knee joint angles and the centre of mass accelerations in three directions.

	TWV 20		TWV 40		TWV 100		TWV 160		TWV 180	
	Tau	Dim	Tau	Dim	Tau	Dim	Tau	Dim	Tau	Dim
Ankle joint angle										
Mean ± SD	34 ± 6.5	7 ± 1.2	21 ± 4.8	8 ± 1.2	11 ± 3.1	6 ± 0.6	8 ± 1.0	5 ± 0.5	8 ± 1.0	5 ± 0.4
Range	21 – 44	6 – 10	13 – 29	6 – 10	8 – 16	5 – 7	7 – 10	5 - 6	7 – 10	5 – 6
Knee joint angle										
Mean ± SD	41 ± 5.0	10 ± 2.1	33 ± 7.5	9 ± 2.0	13 ± 3.3	6 ± 0.7	10 ± 1.4	6 ± 0.7	9 ± 1.2	6 ± 0.8
Range	30 – 49	6 – 14	19 – 45	7 – 14	8 – 19	5 – 7	7 – 12	5 – 7	7 – 11	5 – 7
Centre of mass acceleration anterior-posterior direction										
Mean ± SD	23 ± 5.0	11 ± 2.1	12 ± 3.0	7 ± 1.7	6 ± 1.9	7 ± 0.9	6 ± 1.7	7 ± 0.8	6 ± 1.5	7 ± 0.9
Range	15 – 32	8 – 15	7 – 17	5 – 11	4 – 11	5 – 8	3 – 9	5 – 8	3 – 9	5 – 8
Centre of mass acceleration mediolateral direction										
Mean ± SD	9± 5.1	7 ± 2.1	6 ± 1.7	7 ± 0.9	3 ± 0.4	8 ± 1.2	4 ± 1.8	7 ± 0.8	4 ± 1.6	7 ± 0.8
Range	6 – 24	5 – 11	4 – 8	5 – 8	3 – 4	6 – 10	3 – 9	6 – 8	3 – 8	6 – 9
Centre of mass acceleration vertical direction										
Mean ± SD	13 ± 6.6	7 ± 1.9	5 ± 0.8	8 ± 1.2	5 ± 1.7	6 ± 0.8	5 ± 1.4	6 ± 0.4	5 ± 0.7	6 ± 0.7
Range	4 – 26	5 – 12	4 – 7	6 – 10	3 – 7	5 – 8	3 – 7	5 – 7	4 – 6	5 – 7
